# The relationship between socially-assigned ethnicity, health and experience of racial discrimination for Māori: analysis of the 2006/07 New Zealand Health Survey

**DOI:** 10.1186/1471-2458-13-844

**Published:** 2013-09-13

**Authors:** Ricci B Harris, Donna M Cormack, James Stanley

**Affiliations:** 1 University of Otago, PO Box 7343, Wellington, Wellington South 6242, New Zealand

**Keywords:** Māori, Ethnicity, Socially-assigned ethnicity, Race, Racism, Indigenous, Self-rated health, Psychological distress, New Zealand, Discrimination

## Abstract

**Background:**

In New Zealand, there are significant and long-standing inequalities in a range of health outcomes, risk factors and healthcare measures between Māori (indigenous peoples) and Pākehā (European). This study expands our understanding of racism as a determinant of such inequalities to examine the concept of socially-assigned ethnicity (how an individual is classified by others ethnically/racially) and its relationship to health and racism for Māori. There is some evidence internationally that being socially-assigned as the dominant ethnic group (in this case European) offers health advantage.

**Methods:**

We analysed data from the 2006/07 New Zealand Health Survey for adult participants who self-identified their ethnicity as Māori (n = 3160). The association between socially-assigned ethnicity and individual experience of racial discrimination, and socially-assigned ethnicity and health (self-rated health, psychological distress [Kessler 10-item scale]) was assessed using logistic and linear regression analyses, respectively.

**Results:**

Māori who were socially-assigned as European-only had significantly lower experience of racial discrimination (adjusted odds ratio [OR] = 0.58, 95% confidence interval [CI] = 0.44, 0.78) than Māori who were socially-assigned as non-European. Being socially-assigned as European-only was also associated with health advantage compared to being socially-assigned non-European: more likely to respond with self-rated very good/excellent health (age, sex adjusted OR = 1.39, 95% CI = 1.10, 1.74), and lower Kessler 10 scores (age, sex adjusted mean difference = -0.66, 95% C I = -1.22, -0.10). These results were attenuated following adjustment for socioeconomic measures and experience of racial discrimination.

**Conclusions:**

Results suggest that, in a race conscious society, the way people’s ethnicities are viewed by others is associated with tangible health risk or advantage, and this is consistent with an understanding of racism as a health determinant.

## Background

Internationally, there is substantial evidence of unfair inequalities in health between ethnic groups and, for many countries with histories of colonization, inequalities between indigenous and non-indigenous peoples within the same territory (e.g. New Zealand, Canada, and Australia). In New Zealand, there are significant and long-standing inequalities in a range of health outcomes, risk factors and healthcare between Māori (indigenous peoples) and Pākehā (European) [1-3]. Māori comprise approximately 15% of the New Zealand population [4].

Within New Zealand and internationally, there is recognition of the important role of racism as a basic underlying cause of ethnic inequalities in health [5-10]. Jones defines racism as, “a system (consisting of structures, policies, practices, and norms) that structures opportunity and assigns value based on …the way people look [racially]” [11], p9. Racist practices can operate at the level of institutions and individuals [6,8-13] with a number of pathways through which it may impact health [6,8-13]. Racism can act directly to affect health through trauma and stress pathways, as well as acting indirectly by shaping the distribution of societal resources and individual determinants of health by ethnicity, and influencing access to health care and quality of care by ethnicity [12,13]. In New Zealand, Māori report experiencing disproportionately higher racial discrimination at an individual level that has been linked to a range of adverse health outcomes, heightened health risk and poorer health care as well as contributing to ethnic health inequalities between Māori and Pākehā (European) [5,14,15]. At a structural level, the distribution of socioeconomic resources in New Zealand is highly racialized, with Māori more disadvantaged socioeconomically compared to European [1].

Racialization, or the categorization and stratification of social groups on the basis of ‘race’, is a fundamental element of racism [16]. ‘Race’ or ‘ethnicity’ is increasingly understood as socially constructed, with official measurement of ethnicity in New Zealand now based on self-identified cultural affiliation, following a shift over time from more biological based definitions [17]. However, in racialized societies, race/ethnicity remains a salient externally-imposed category that continues to draw on discredited notions of biology, blood and genes [17]. This study examines the concept of socially-assigned ethnicity and its relationship to health for Māori. Socially-assigned (or socially-ascribed) race/ethnicity refers to the way in which an individual is classified by others racially (or ethnically), based on supposed ‘racial’ markers, principally physical appearance [18]. The way people are racially or ethnically viewed by others is linked to how they are treated and the opportunities they receive in society. Jones [11] suggests that it is socially-assigned race that results in health impacts, rather than self-identified race *per se*. There has been some initial support for this hypothesis, with a study using data from the United States 2004 Behavioral Risk Factor Surveillance System (BRFSS) showing that being socially-assigned as ‘white’ confers a health advantage, irrespective of one’s self-identified ethnicity [18].

Our study uses data from the 2006/07 New Zealand Health Survey to examine health among Māori participants, specifically the association of socially-assigned ethnicity with health and with individual experiences of racism amongst Māori. We hypothesize that among people who self-identify as Māori, being socially-assigned as of the dominant ethnic group (‘European’) will be associated with health advantage. We hypothesize that this will operate in part due to lower exposure to racial discrimination among people socially-assigned as European.

## Methods

### Study design

The study uses data from the 2006/07 New Zealand Health Survey (for adult respondents). This is the fourth national population based health survey conducted by the New Zealand Ministry of Health. It provides nationally representative information on measures of self-reported health, health service utilization, health risk and protective factors, and demographic variables among the usually resident population aged 15 years and over [19].

#### Survey design

The survey uses an area based sampling frame with meshblocks (small geographical areas of approximately 100 people) as the primary sampling units. Sampling was undertaken using a multi-stage, stratified, probability-proportional-to-size design. 1385 meshblocks were initially selected, followed by dwellings within each meshblock and finally one eligible adult from each selected dwelling. Increased sampling of Māori, Pacific and Asian peoples (relative to population size) was achieved mostly through the use of a large screening sample, and to a limited extent through disproportionate sampling of areas with higher proportions of Māori at the District Health Board level. A total of 12488 adults were surveyed in face-to-face interviews between 6 October 2006 and 29 November 2007. This included 3160 people who self-identified as Māori (either alone or in combination with other ethnicities). The weighted response rate was 67.9% overall and 67.5% for Māori. Further detail on the survey design can be found elsewhere [19].

Ethics approval for the study reported in this paper was granted by the New Zealand Health and Disability Multi-Region Ethics Committee (MEC/10/050/EXP).

### Key variables

#### Self-identified ethnicity

This study is restricted to those people who self-identified as Māori (either alone or in combination with another ethnicity, n = 3160). In New Zealand, ethnicity is officially conceptualized as a social construct of group affiliation and cultural identity. The term ‘ethnicity’ is used rather than ‘race’ in the collection of official statistics with standard protocols in the health sector for the collection, recording and outputting of ethnicity data for statistical purposes [20]. The 2006/07 NZHS uses the same ethnicity question as the New Zealand Census. This asks people to identify the ethnic group or groups they feel they belong to. People can identify with more than one ethnic group. A number of ethnic groups are given as response options as well as an ‘other’ option that allows people to write their ethnicity. In line with indigenous rights to self-identification [21], we considered Māori as anyone who self-identified as Māori, either alone or in combination with another ethnicity. It is standard practice to present population health statistics and ethnic inequalities by self-identified ethnicity in New Zealand [20].

#### Socially-assigned ethnicity

In addition to self-identified ethnicity, participants were also asked to report their socially-assigned ethnicity. Participants were asked, “Earlier you told me your ethnicity. Now I will ask you some questions about reactions to your ethnicity. How do other people usually classify you in New Zealand?” Response options were the same as for the self-identified ethnicity question and people could respond with multiple ethnicities. The socially-assigned ethnicity question was based on a similar question developed for the ‘Reactions to race’ module of the Behavioral Risk Factor Surveillance System (BRFSS) in the United States [22].

In order to test the hypothesis that being socially-assigned as dominant will be associated with health advantage and lower exposure to racial discrimination, socially-assigned ethnicity responses were grouped into dominant (European-only, n = 771) and non-dominant (any socially-assigned Māori or other non-European ethnicity, either alone or in combination with socially-assigned European ethnicity, n = 2389) groups. This approach is similar to that of other studies on the impact of socially-assigned ethnicity on health [18,23].

#### Racial discrimination

People were asked about their individual experience of racial discrimination in New Zealand in five settings. These were experience of an ethnically motivated verbal attack, physical attack, unfair treatment because of ethnicity by a health professional, unfair treatment in work or gaining a job, and unfair treatment when renting or buying housing. Prevalence of ‘ever’ experiencing racial discrimination for each of these domains was analysed. For the purposes of logistic regression modelling, these racial discrimination items were reduced to a three level variable when used as an explanatory variable (i.e. never, one domain ever, two domains or more ever), or a binary variable of any experience of racial discrimination ever when modelling experience of racism as an outcome (yes/no). Additional information on development, use and variable reduction of the racial discrimination questions can be found elsewhere [24].

#### Health outcomes

Two health measures were examined, general self-rated health [3] and non-specific psychological distress (using the Kessler 10-item scale) [25].

In terms of general self-rated health, participants were asked, ‘In general, would you say that your health is: excellent, very good, good, fair or poor’. In order to assess health advantage related to socially-assigned ethnicity, responses were dichotomized to excellent/very good versus good/fair/poor. This is in line with the two similar studies in this area [18,23]. General self-rated health has been shown to be a valid indicator of health status with strong evidence it is predictive of long term illness and mortality [26,27].

Psychological distress was measured using the Kessler 10-item scale. This scale asks 10 questions on how frequently participants’ felt symptoms of psychological distress in the past four weeks. Responses range from ‘None of the time’ to ‘all of the time’ on a five-point scale (0 to 4 per item). An overall score is generated from 0 to 40 with higher scores reflecting higher levels of psychological distress [3,25,28]. K10 scores were treated as a continuous outcome variable in these analyses [29].

#### Other covariates

Other variables adjusted for in multivariable analyses included gender (men, women (reference)) and age group (15—24 (reference), 25—34, 35—44, 45—54, 55—64, 65—74, 75+ years).

Four measures of socioeconomic position were also adjusted for in regression analyses. These included education qualification, living standards, individual level deprivation and area-based deprivation. Education qualification was classified as no secondary qualification attained (reference) and attained secondary qualification or higher.

Living standards were measured using the Economic Living Standards Index short form (ELSI-SF). This scale includes 25 items that cover questions on the three conventional constructs of living standards measures: economizing behaviours, ownership of durable assets, and social participation restrictions. Scores range from 0—31 and are classified into seven categories of living standards: very good, good, comfortable (reference), fairly comfortable, some hardship, significant hardship, and severe hardship [30,31].

Individual level deprivation was measured using the New Zealand Index of Socioeconomic Deprivation for Individuals (NZiDep) [32]. The index comprises 8 questions that are scored to create a five-point individual-level index of socioeconomic deprivation. Questions ask individuals about experiencing particular situations in the last 12 months e.g. being forced to buy cheaper food, being out of paid work, receiving a benefit, putting up with feeling cold to save on heating costs. Scores range from 1 (no deprivation characteristics [reference]), 2 (one characteristic), 3 (two characteristics), 4 (three or four characteristics) and 5 (five or more deprivation characteristics).

Area deprivation was measured using the New Zealand Index of Deprivation 2006 (NZDep2006). This provides a deprivation score for each meshblock (small areas of approximately 100 people) in New Zealand. It was created by combining (by principal component analysis) nine variables from the 2006 Census (benefit receipt, house-hold income, living in a single-parent family, home ownership, employment status, access to a telephone, qualifications, living space, and access to a car). Scores are categorized into deciles (1 (ref) —10) with higher scores indicating greater deprivation [33].

### Data analysis

We analysed data in SAS 9.2 (SAS Institute, NC) using Survey analysis based procedures. All analyses account for the stratification and clustering elements of the survey design, with the exception of the descriptive statistics covering the cross-tabulation of self-identified and socially-assigned ethnicity. All data are weighted for probability of selection and non-response using survey weights to produce representative estimates for the New Zealand adult population and for appropriate calculation of confidence intervals.

Unweighted frequencies and weighted prevalence of demographic variables, experience of racial discrimination and self-rated health outcomes were calculated for people socially-assigned as dominant and non-dominant ethnicity. Weighted means, medians and interquartile ranges were estimated for K10 scores.

Logistic regression (with the Proc Surveylogistic procedure in SAS) was used to examine the independent association of socially-assigned ethnicity with any experience of racial discrimination ever (as a binary variable). Unadjusted and sequentially adjusted models (adding new adjustment covariates to the preceding model) are presented. Model covariates were chosen prior to analysis, and include age, gender, and socioeconomic position (qualification, ELSI, NZiDep, NZDep2006).

Logistic regression (with the Proc Surveylogistic procedure in SAS) was also used to examine the independent association of socially-assigned ethnicity with optimal self-rated general health (excellent or very good self-rated health). Unadjusted and sequentially adjusted models are presented. Covariates again include age, gender, socioeconomic position (qualification, ELSI, NZiDep, NZDep2006) and individual experience of racial discrimination ever (in three levels).

Modelling of K10 scores was conducted using Proc Surveyreg, using a linear model approach. Model covariates, and the sequential sets of covariates adjusted for at each stage of modelling, were the same as described for the self-rated health analysis above. K10 scores in the general population are known to be right skewed rather than normally distributed, which might generally suggest that linear regression on raw data is not appropriate. However, group comparison of means are still valid for these data, as the central limit theorem dictates that in a suitably large sample the standard error will be a valid estimate of sampling variation processes, and by extension confidence intervals and hypothesis tests of regression parameters, will also be valid [34]. These methods have been applied previously to analysis of K10 data from a complex survey in New Zealand [29].

In the models examining the association between socially-assigned ethnicity and health (general self-rated health and psychological distress), age and sex were adjusted for as potential confounders, while socioeconomic measures and racial discrimination were adjusted for as markers of institutional and individual racial discrimination and potential pathway variables by which racial discrimination based on socially-assigned ethnicity may be operating to affect health. Reductions in ORs or mean differences after adjustment for each set of variables would be consistent with this theory.

#### Self-identified ethnicity combinations

All regression models with health and experience of racial discrimination as outcomes were also undertaken with self-identified ethnicity (reported both European and Māori ethnicity, versus reported Māori ethnicity but not European) included in addition to socially-assigned ethnicity to test whether health and racial discrimination outcomes were associated with self-identified ethnicity above and beyond the association seen with socially-assigned ethnicity.

Self-identified ethnicity was not related to outcomes after accounting for socially-assigned ethnicity and so was not included in the final models (data not shown). Examination of point estimates for the socially-assigned ethnicity variable in these models indicated that the inclusion of the different Māori self-identified categories did not affect the relationship between socially-assigned ethnicity and the outcome variables of interest.

Interaction terms between the new self-identified ethnicity and the socially assigned ethnicity (coded as per the main analysis) were also examined: these interaction terms were not significant for the health outcomes (suggesting that the impact of socially assigned ethnicity does not depend on self-identified ethnicity; interaction term likelihood ratio test p-values = 0.681 and 0.512, for SF-36 and K-10 outcomes respectively). For the racial discrimination outcome, this interaction was significant (LR test p-value = 0.002,) and this relationship is described more fully in the Results section.

## Results

Of the 12488 participants in the 2006/07 New Zealand Health Survey, 3160 self-identified Māori as one of their ethnic groups. Among this Māori sample, Table 1 shows the four most common self-identified ethnicity combinations (98.7%, n = 3118) with the remaining Māori ethnic combinations grouped together as Māori/Other (1.3%, n = 42). Within each of these self-identified combinations, Table 1 also shows the most common socially-assigned ethnicity combinations. Among Māori included in non-European socially-assigned ethnic groups, most (2240 of 2389, 94%) were socially-assigned as Māori either alone or in combination with other ethnic groups while 149 (6%) were socially-assigned as another non-Māori/non-European ethnicity group.

**Table 1:** Cross tabulation of self-identified and socially-assigned ethnicity combinations among people who self-identify as Māori

	**Socially-assigned ethnicity n (% within self-identified ethnicity row)**													
**Self-identified ethnicity**	**M**		**M, E**		**M, P**		**M,O**		**E**		**P**		**O**	
M (n = 1660)	1327	(79.9)	64	(3.9)	44	(2.7)	27	(1.6)	113	(6.8)	45	(2.7)	40	(2.4)
M, E (n = 1351)	438	(32.4)	205	(15.2)	13	(1.0)	16	(1.2)	635	(47.0)	14	(1.0)	30	(2.2)
M, P (n = 58)	38	(65.5)	0		8	(13.8)	1	(1.7)	1	(1.7)	8	(13.8)	2	(3.4)
M, P, E (n = 49)	23	(46.9)	1	(2.0)	6	(12.2)	3	(6.1)	12	(24.9)	1	(2.0)	3	(6.1)
M, O (n = 42)	22	(52.4)	2	(4.8)	1	(2.4)	1	(2.4)	10	(23.8)	0		6	(14.3)

*M*=Māori, *P*=Pacific, *E*=European, *O*=other ethnicity or ethnic combinations including Asian.

Agreement between self-identified and socially-assigned ethnic groups was varied. The two most common self-identified ethnicity combinations among the study group were Māori-only (n = 1660) and Māori/European (n = 1351). Among participants who self-identified as Māori-only, 80% reported their socially-assigned ethnicity as Māori-only, while 7% reported being socially-assigned as European-only and 4% as both. Among those who self-identified as both Māori and European, 32% were socially-assigned as Māori-only, 47% as European-only and 15% as both Māori and European.

Table 2 shows the weighted prevalences of demographic variables for Māori participants socially-assigned as European-only, and those socially-assigned to any Māori or other non-European ethnicity. These groups had similar age and sex profiles. However, Māori who were socially-assigned as being European-only tended to be more advantaged with regards to socioeconomic measures than Māori socially-assigned as being any Māori or other non-European ethnicity. Māori socially-assigned as European-only were more likely to have at least a secondary educational qualification, report higher living standards, be less socioeconomically deprived at an individual level (NZiDep) and also tended to live in less deprived areas (NZDep decile) than Māori who were socially-assigned to Māori or non-European ethnic groups (Table 2).

**Table 2:** Distribution of demographic variables by socially-assigned ethnicity combinations among people who self-identify as Māori

	**Socially-assigned ethnicity**	
**Characteristic**	**European-only n = 771**	Any Māori or other non-European ethnicities* n = 2389
	**Unweighted frequency (weighted %)**	**Unweighted frequency (weighted %)**
**Gender**		
Female	484 (54.5)	1471 (53.3)
Male	287 (45.5)	918 (46.7)
**Age group**		
15–24 years	162 (27.7)	482 (27.0)
25–34 years	172 (21.6)	553 (20.8)
35–44 years	166 (21.3)	504 (20.5)
45–54 years	108 (13.6)	413 (16.6)
55–64 years	83 (9.2)	223 (8.7)
65–74 years	52 (4.5)	150 (4.8)
75+ years	28 (2.1)	64 (1.6)
**Qualification**		
No secondary	279 (32.6)	1183 (48.5)
Secondary education	492 (67.4)	1198 (51.5)
**ELSI**		
Severe hardship	23 (1.9)	111 (4.0)
Significant hardship	31 (4.8)	144 (5.4)
Some hardship	49 (5.6)	226 (8.5)
Fairly comfortable	85 (8.4)	335 (14.7)
Comfortable	183 (25.6)	539 (23.7)
Good	283 (39.5)	783 (33.9)
Very good	102 (14.2)	206 (9.8)
**Area deprivation (NZDep06)**		
Decile 1 (least deprived)	53 (7.9)	42 (2.1)
Decile 2	50 (6.8)	102 (4.8)
Decile 3	63 (9.0)	86 (3.6)
Decile 4	80 (10.7)	118 (6.0)
Decile 5	64 (8.0)	144 (6.5)
Decile 6	91 (10.1)	204 (8.5)
Decile 7	96 (13.5)	251 (10.9)
Decile 8	100 (13.3)	273 (12.2)
Decile 9	71 (10.9)	438 (17.8)
Decile 10 (most deprived)	103 (9.9)	731 (27.5)
**Individual deprivation (NZiDep)**		
Level 1 (least deprived)	408 (54.3)	988 (43.0)
Level 2	160 (22.1)	483 (21.5)
Level 3	71 (8.6)	309 (12.6)
Level 4	84 (10.2)	378 (15.2)
Level 5 (most deprived)	48 (4.9)	226 (7.7)

*Any socially-assigned Māori or other non-European ethnicity, either alone or in combination with socially-assigned European ethnicity (1848 Māori only, 288 Māori and European, 105 Māori and non-European, 148 other socially-assigned combinations).

Univariate analyses of socially-assigned ethnicity and key variables of individual experience of racial discrimination and self-rated health showed that being socially-assigned as European-only tended to be associated with lower reporting of experience of racial discrimination and better self-rated health than being socially-assigned to any non-European group, including Māori (Table 3). People socially-assigned as European-only tended to have lower mean psychological distress scores than those who were socially-assigned as non-European.

**Table 3:** Distribution of key variables by socially-assigned ethnicity combinations among people who self-identify as Māori

**Characteristic**	**Socially-assigned ethnicity**	
	**European-only**	Any Māori or other non-European ethnicities*
	**Unweighted frequency**	**Unweighted frequency**
	**weighted % (95% CI)**	**weighted % (95% CI)**
**Racial discrimination (reported individual experience)**		
Verbal assault (ever)	147, 18.2% (14.5, 21.8)	578, 23.7% (21.5, 25.9)
Physical assault (ever)	27, 3.4% (1.6, 5.2)	152, 6.1% (4.9, 7.3)
Discrimination in health (ever)	29, 3.4% (1.8, 4.9)	147, 5.6% (4.5, 6.6)
Discrimination in work (ever)	19, 1.9% (0.8, 3.1)	169, 6.6% (5.4, 7.8)
Discrimination in housing (ever)	22, 2.2% (1.0, 3.3)	235, 8.1% (6.9, 9.4)
No experiences	594, 78.2% (74, 82.4)	1583, 67.7% (65.3, 70.1)
One experience	123, 16.3% (12.8, 19.8)	474, 20.0% (18.1, 22.0)
Two or more	50, 5.5% (3.4, 7.6)	319, 12.2% (10.7, 13.8)
**Health outcome**		
**Self-rated health**		
Excellent self-rated health	113, 12.9% (9.9, 16.0)	305, 13.9% (12.0, 15.8)
Very good self-rated health	310, 44.4% (39.5, 49.3)	861, 35.4% (32.9, 37.8)
Good self-rated health	265, 32.6% (27.9, 37.2)	857, 35.9% (33.5, 38.3)
Fair self-rated health	63, 7.4% (5.2, 9.6)	295, 12.2% (10.6, 13.8)
Poor self-rated health	20, 2.7% (1.2, 4.2)	71, 2.7% (1.9, 3.4)
**Kessler Psychological Distress Scale (K10)**		
Mean (95% CI)	4.12 (3.66, 4.58)	4.77 (4.47, 5.06)
Median (Interquartile range)	2.1 (0.2, 5.0)	2.2 (0.2, 6.2)

Note: unweighted frequencies; weighted prevalences, means, medians and 95% CIs.

*Any socially-assigned Māori or other non-European ethnicity, either alone or in combination with socially-assigned European ethnicity (1848 Māori only, 288 Māori and European, 105 Māori and non-European, 148 other socially-assigned combinations).

Māori who were socially-assigned as being European-only were significantly less likely to report experience of racial discrimination (Table 4; OR = 0.59, 95% CI = 0.45, 0.76). Sequential adjustment for sociodemographic and socioeconomic factors had little impact on this estimate (fully adjusted OR = 0.58, 95% CI = 0.44, 0.78). A significant interaction suggested that this association depended upon the respondent’s self-identified ethnicity. For those who self-identified as Māori and European, the impact of being socially-assigned as European was protective of experiencing racial discrimination (OR for socially-assigned ethnicity = 0.49, 95% CI = 0.35, 0.68); for those who self-identified as Māori with no European ethnicity, the impact on experience of racial discrimination of being socially assigned as European was close to null (OR = 1.07, 95% CI = 0.6, 1.88). These odds ratios are from the unadjusted model; the interaction followed a similar pattern in the fully-adjusted model.

**Table 4:** Odds ratios from logistic regression models for reported individual experience of racial discrimination (ever)

	**Socially-assigned ethnicity, OR for experience of racism (95% CI)**	
**Adjusted estimate source**	Any Māori or other non-European ethnicities*	**European-only**
**Unadjusted model**	Ref	0.59 (0.45, 0.76)
**Adjusted models (sequential)**		
+ age, sex	Ref	0.59 (0.45, 0.77)
+ qualification	Ref	0.58 (0.44, 0.75)
+ ELSI, NZiDep, NZDep06	Ref	0.58 (0.44, 0.78)

*Includes reporting any socially-assigned non-European ethnicity (largely Māori) either alone or in combination with socially-assigned European ethnicity. + indicates adding new adjustment covariates to the preceding model.

In multivariable analysis, Māori who were socially-assigned as European-only had a significant health advantage (reported excellent or very good health) compared to other Māori after adjusting for age and sex (OR = 1.39, 95% CI = 1.10, 1.74; Table 5). This appeared to be due to the socioeconomic advantage and lower reporting of racial discrimination experiences associated with being socially-assigned as European-only (Adjusting for experience of racial discrimination immediately after adjusting for age and sex slightly attenuated this association, OR = 1.33, 95% CI = 1.05, 1.68 [not shown in table]). The difference between Māori socially-assigned as European-only and those assigned as any non-European group (including Māori) was attenuated after the sequential adjustment of socioeconomic measures (OR = 1.15, 95% CI 0.90, 1.46) and racial discrimination (OR = 1.11, 95% CI = 0.87, 1.42).

**Table 5:** Odds ratios from logistic regression models for excellent/very good health (compared to good/fair/poor health)

	**Socially-assigned ethnicity, OR (95% CI)**	
**Adjusted estimate source**	Any Māori or other non-European ethnicities*	**European-only**
**Unadjusted model**	Ref	1.39 (1.10, 1.74)
**Adjusted for age, sex**	Ref	1.39 (1.10, 1.74)
**Adjusted for potential pathway variables**		
+ qualification	Ref	1.28 (1.02, 1.61)
+ ELSI, NZiDep, NZDep06	Ref	1.15 (0.90, 1.46)
+ Racism (individual experience)	Ref	1.11 (0.87, 1.42)

*Includes reporting any socially-assigned non-European ethnicity (largely Māori) either alone or in combination with socially-assigned European ethnicity. + indicates adding new adjustment covariates to the preceding model.

Analysis of difference in mean K10 scores also showed that Māori who were socially-assigned as any Māori or non-European ethnic group had significantly higher psychological distress scores in both the unadjusted and the age/sex adjusted models compared to Māori who were socially-assigned as European-only. This appeared to operate via socioeconomic advantage and lower experience of racial discrimination among Māori socially-assigned as European-only, with differences in mean K10 scores attenuated after adjusting for these variables (Table 6). Adjusting for experience of racial discrimination immediately after adjustment for age and sex had a considerable attenuating effect on the estimate of K10 group difference (mean difference = 0.35, 95% CI = −0.89, 0.20 [not shown in table]).

**Table 6:** Linear regression estimates for difference in mean K10 score

	**Socially-assigned ethnicity**	
**Adjusted estimate source**	Any Māori or other non-European ethnicities*	**European-only ethnicity**
		**Mean difference (95% CI)**
**Unadjusted model**	Ref	-0.62 (-1.19, -0.05)
**Adjusted for age, sex**	Ref	-0.66 (-1.22, -0.10)
**Adjusted for potential pathway variables**		
+ education	Ref	-0.52 (-1.08, 0.03)
+ ELSI, NZiDep, NZDep06	Ref	-0.17 (-0.65, 0.32)
+ Racism (individual experience)	Ref	0.04 (-0.44, 0.51)

*Includes reporting any socially-assigned non-European ethnicity (largely Māori) either alone or in combination with socially-assigned European ethnicity. + indicates adding new adjustment covariates to the preceding model.

## Discussion

Our study builds on literature examining socially-assigned race/ethnicity and health, and suggests that ethnic appearance is an important determinant of health, for Māori at least. We found that among the self-identified Māori population, Māori who reported being socially-assigned as European-only had a health advantage compared with those who were socially-assigned as Māori and/or any other non-European group. Māori who were socially-assigned as European-only were significantly more likely to report their health as excellent or very good and have lower levels of psychological distress than other Māori. This relationship was attenuated after adjusting for socioeconomic measures and individual experience of racial discrimination (and no longer statistically significant after adjustment). The relationship between socially-assigned ethnicity and health was independent of self-identified ethnic group combinations.

Our findings are consistent with those of Jones et al. who analysed data from a large United States study of participants from eight states using the Reactions to Race module of the 2004 BRFSS [18]. Jones et al. found that being classified by others as ‘White’ was associated with health advantage even among participants who did not self-identify as ‘White’ and included ‘Hispanic’, ‘American Indian’ and ‘people who identified with more than one race’ [18]. The authors conclude that this reflects the effects of racism on health in the United States, not only in terms of disadvantage, but also the advantages of ‘whiteness’ in a race conscious society [18]. In contrast to Jones’ findings, analysis of the Michigan/Wisconsin BRFSS data found that there was not a health advantage associated with being socially-assigned to the White group for (self-identified) non-White groups, and that self-assessment of race/ethnicity predicted health status better than socially-assigned race/ethnicity [23]. The authors suggest that because of the systemic and societal nature of racism, and the segregated histories of the particular communities they investigated, “…there may be little opportunity for White privilege to aggregate to the community level, and thus the potential privilege from being socially-assigned as White may be partially or wholly un-realized”. Our study expands on the analyses of Jones’ et al. [18] and Ridings et al. [23] to also examine the association between socially-assigned ethnicity and reported individual experience of racism, with assignment to the dominant European ethnic group significantly related to reduced exposure to individual level racial discrimination, as well as exploring an additional health measure of psychological distress. It also provides evidence in a different social context and for an indigenous population.

Other studies have also examined health and social differentials within the Māori population and shown that health and socioeconomic differences exist for different Māori populations based on their self-identified ethnicity. For example, people who identify solely as Māori have been shown to have more disadvantaged socioeconomic status and worse health than people who identify as Māori and European [35-38]. Various hypotheses have been posited to explain differences in the ways Māori self-identify their ethnicity or ethnicities that may also be linked to more or less disadvantage. These include ‘cultural’ affiliation or strength of identity reasons [35] as well as skin colour and appearance [39]. Kukutai [35] notes that “orientation towards the European mainstream confers benefits in terms of better outcomes” (p 100). Our study suggests that these types of analyses may have been confounded by socially-assigned ethnicity. The relationship between socially-assigned ethnicity and health was unchanged when Māori self-identified categories were added to the models (people self-identifying as Māori alone or in combination with another minority ethnic group compared with people identifying as Māori and European). Māori self-identified categories were not associated with health when adjusted for socially-assigned ethnicity.

The relationship between socially-assigned ethnicity and health appears to operate via more advantaged socioeconomic position and reduced exposure to individual experience of racism. It is acknowledged that racism plays a fundamental role in structuring social and economic ethnic disparities [8]. The relationship between socially-assigned ethnicity, socioeconomic position and individual experience of racism in our study, while not definitive, is consistent with racism operating at both the institutional and individual levels. In our analysis, socioeconomic status and individual experience of racial discrimination are both viewed as markers of racism and potential pathway indicators by which racial discrimination based on socially-assigned ethnicity may be operating to affect health.

This is the first time socially-assigned ethnicity has been analysed from the New Zealand Health Survey and provides further insight into intra-group health differences within the Māori population. It expands on the few international studies in the area to directly demonstrate the link between socially-assigned ethnicity and individual experience of racism and examines an additional health measure in psychological distress. A major study strength is the ability to provide nationally representative information for Māori. In addition, this study examines health advantage, rather than disadvantage, and how privilege may be afforded to people based on their appearance as European, even when they self-identify as a different ethnicity. The measure of socially-assigned ethnicity may reflect additional aspects of racial discrimination that are not fully captured by self-report of individual experiences [18], which can be limited by peoples’ willingness and ability to report them [6]. In our study, adjusting for individual experience of racial discrimination attenuated the differences in health between the socially-assigned groups. While this was to a limited degree, the questions on individual experience of racism were only asked in a very specific number of situations and may well underestimate people’s actual exposure [5].

There are also some important limitations to consider in the interpretation of our study findings. Our study is restricted to the Māori population, and so cannot be generalized to consider the effect of socially-assigned ethnicity on other groups and on ethnic inequalities between groups, although further work is underway to examine this, including how socially-assigned ethnicity impacts on the health of people who self-identify as European or other ethnic groups. The measures are self-reported with recognised limitations of such measures in health and racism literature, including the role of social desirability that has the potential to impact on reporting of ethnicity, discrimination and health measures in this study [6]. The New Zealand Health Survey is a cross-sectional survey, and so usual caveats on attributing causality apply, particularly temporality. For example, it is possible that experience of racial discrimination may enforce the feeling of being classified as non-European: thus self-reported socially-assigned ethnicity could potentially be influenced by experience of racial discrimination.

There are some other potential issues with the measure of socially-assigned ethnicity. While it is intended to capture how other people view a person’s ethnicity, it is self-reported socially-assigned ethnicity, and not observer reported [40]. Unlike the United States, where multiple ethnicities are not commonly reported, multiple reporting of self-identified ethnic groups in New Zealand is not uncommon. While the wording of the socially-assigned question implies a single response, “How do other people usually classify you?”, multiple responses were allowed and were fairly common, although not to the same extent as the self-identified question. This complicates the analysis and also the interpretation of multiple socially-assigned ethnic groups as it is unclear whether a person is sometimes seen as one ethnicity and sometimes another, or whether they may be viewed as both ethnic groups simultaneously. In addition, the context a person is in may influence their responses to this question e.g. they may be viewed as different ethnic groups in different contexts [40-42]. As the question is trying to capture the ethnic group that other people recognize and react to in wider society, a more context specific question with single reporting options may be better. For example, in Australia, a similar question for use in the indigenous population asks, “Do people you meet for the first time know that you are Indigenous?” capturing both context and a single response [43].

While this study cannot definitely say that socially-assigned ethnicity reflects differential experiences of racism on health, it is consistent with this theory. Our study does not examine the specific mechanisms through which socially assigned ethnicity impacts on health for Māori and there are likely to be a number of factors through which this may occur including associations with possible individual risk and protective factors and interactions and experiences with health services.

Finally, the use of excellent or very good health to ascertain health advantage is not the usual cut point for analysis of the general self-rated health question. This cut point was used to examine optimal health both from a theoretical perspective in order to explore health advantage rather than disadvantage and also for comparability with other studies in the field of socially-assigned ethnicity and health [18,23].

Jones et al. [18] notes the importance of measuring both socially-assigned ethnicity and self-identified ethnicity in order to better understand how race and ethnicity influence health. In New Zealand for example, self-identified ethnicity remains a key measure of ethnic inequalities, with socially-assigned ethnicity a potentially stronger marker of risk for exposure to racism [44] rather than a measure of how an individual or group necessarily views or expresses their ethnic identity. For Māori and other indigenous peoples, socially-assigned ethnicity is unlikely to fully capture the Māori population who have particular indigenous or treaty rights, including the right to self-determination of their identity and the right to monitor Crown action and inaction in relation to Māori health and other outcomes [1,17,35]. Socially-assigned ethnicity does, however, provide insight into the determinants of health for Māori and potentially for other ethnic groups in New Zealand and more broadly.

## Conclusions

Results of this study suggest that, in a race conscious society, the way people’s ethnicities are viewed by others appears to have tangible health risk or advantage, and this is consistent with an understanding of racism as a health determinant. Dismantling the structures of racism is complex yet vital in our efforts to achieve a fair society that facilitates equitable outcomes in health and other social indicators and also enables self-determination of priorities and solutions for Māori.

## Abbreviations

CI: Confidence interval; ELSI: Economic living standards index short form; Ethnic groups (M: Māori; P: Pacific; E: European; O: other ethnicity or ethnic combinations including Asian); K10: Kessler 10-item scale; NZDep06: New Zealand Index of Deprivation 2006; NZiDep: New Zealand Index of Socioeconomic Deprivation for Individuals; OR: Odds ratio; Ref: reference group.

## Competing interests

The authors declare that they have no competing interests.

## Authors’ contributions

RH and DC conceived the study. All authors participated in the study design and interpretation of findings. JS undertook the data analysis. RH led the initial drafting of the manuscript. DC and JS contributed to the writing of subsequent versions. All authors have read and approved the final version of the manuscript. The results presented in this paper are the work of the authors.

## Authors’ information

RH (MB ChB, MPH, FNZCPHM), senior research fellow.

DC (PhD), senior research fellow.

JS (PhD), senior research fellow and biostatistician.

## Pre-publication history

The pre-publication history for this paper can be accessed here:

http://www.biomedcentral.com/1471-2458/13/844/prepub

## References

[B1] Robson B, Harris R Hauora: Māori Standards of Health IV. A study of the years 2000–2005 2007 Wellington: Te Rōpū Rangahau Hauora a Eru Pōmare

[B2] Tobias M Blakely T Matheson D Rasanathan K Atkinson J Changing trends in indigenous inequalities in mortality: lessons from New Zealand Int J Epidemiol 2009 38 1711 1722 10.1093/ije/dyp156 19332501

[B3] Ministry of Health A Portrait of Health. Key Results of the 2006/07 New Zealand Health Survey 2008 Wellington: Ministry of Health

[B4] Estimates and projectionshttp://www.stats.govt.nz/browse_for_stats/population/estimates_and_projections.aspx

[B5] Harris R Cormack D Tobias M Yeh L-C Talamaivao N Minster J Timutimu R The pervasive effects of racism: experiences of racial discrimination in New Zealand over time and associations with multiple health domains Soc Sci Med 2012 74 2012 408 415 22204840 10.1016/j.socscimed.2011.11.004

[B6] Krieger N Methods for the scientific study of discrimination and health: an ecosocial approach Am J Public Health 2012 102 5 936 944 10.2105/AJPH.2011.300544 22420803 PMC3484783

[B7] Ministry of Health Reducing inequalities in health 2002 Wellington: Ministry of Health

[B8] Nazroo JY The structuring of ethnic inequalities in health: economic position, racial discrimination, and racism Am J Public Health 2003 93 2 277 284 10.2105/AJPH.93.2.277 12554585 PMC1447729

[B9] Williams DR Race and health: basic questions, emerging directions Ann Epidemiol 1997 7 5 322 333 10.1016/S1047-2797(97)00051-3 9250627

[B10] Tobias M Harris R Racism and health in New Zealand Ethn Health 2009 15 4 318 320 21171223

[B11] Jones CP Confronting institutionalized racism Phylon 2002 150 7 22

[B12] Jones CP Invited commentary: “race,” racism, and the practice of epidemiology Am J Epidemiol 2001 154 4 299 304 10.1093/aje/154.4.299 11495851

[B13] Krieger N Berkman L, Kawachi I Discrimination and health Social epidemiology 2000 New York: Oxford University Press 36 75

[B14] Harris R Cormack D Tobias M Yeh L-C Talamaivao N Minster J Timutimu R Self-reported experience of racial discrimination and health care use in New Zealand: results from the 2006/07 New Zealand health survey Am J Public Health 2012 102 1012 1019 10.2105/AJPH.2011.300626 22420811 PMC3483923

[B15] Harris R Tobias M Jeffreys M Waldegrave K Karlsen S Nazroo J Effects of self-reported racial discrimination and deprivation on Māori health and inequalities in New Zealand: cross-sectional study Lancet 2006 367 9527 2005 2009 10.1016/S0140-6736(06)68890-9 16782491

[B16] Garner S Racisms: an introduction 2010 London: Sage Publications

[B17] Cormack D The practice and politics of counting: ethnicity data in official statistics in Aotearoa/New Zealand 2010 Wellington: Te Rōpū Rangahau Hauora a Eru Pōmare

[B18] Jones CP Truman BI Elam-Evans LD Jones CA Jones CY Jiles R Rumisha SF Perry GS Using “socially assigned race” to probe white advantages in health status Ethn Dis 2008 18 4 496 504 19157256

[B19] Ministry of Health Methodology Report for the 2006/07 New Zealand Health Survey 2008 Wellington: Ministry of Health

[B20] Ministry of Health Ethnicity Data Protocols for the Health and Disability Sector 2004 Wellington: Ministry of Health

[B21] UN General Assembly, United Nations Declaration on the Rights of Indigenous Peoples: resolution/adopted by the General Assembly, 2 October 2007, A/RES/61/295 http://www.un.org/esa/socdev/unpfii/documents/DRIPS_en.pdf

[B22] Centers for Disease Control and Prevention (CDC) Risk Factor Surveillance System Survey Questionnaire 2004 Atlanta, Georgia: U.S. Department of Health and Human Services, Centers for Disease Control and Prevention

[B23] Ridings C Rafferty A Weir S ‘Reactions to Race: analysis from Michigan and Wisconsin’. Michigan BRFSS/Health Disparities Surveillance Brief 2010 Lansing, MI: Michigan Department of Community Health, Chronic Disease Epidemiology Section

[B24] Harris R Tobias M Jeffreys M Waldegrave K Karlsen S Nazroo J Racism and health: the relationship between experience of racial discrimination and health in New Zealand Soc Sci Med 2006 63 6 1428 1441 10.1016/j.socscimed.2006.04.009 16740349

[B25] Kessler RC Barker PR Colpe LJ Epstein JF Gfroerer JC Hiripi E Howes MJ Normand SL Manderscheid RW Walters EE Screening for serious mental illness in the general population Arch Gen Psychiatry 2003 60 2 184 189 10.1001/archpsyc.60.2.184 12578436

[B26] Idler EL Benyamini Y Self-rated health and mortality: a review of twenty-seven community studies J Health Soc Behav 1997 38 1 21 37 10.2307/2955359 9097506

[B27] DeSalvo KB Bloser N Reynolds K He J Muntner P Mortality prediction with a single general self-rated health question. A meta-analysis J Gen Intern Med 2006 21 3 267 275 10.1111/j.1525-1497.2005.00291.x 16336622 PMC1828094

[B28] Andrews G Slade T Interpreting scores on the Kessler psychological distress scale (K10) Aust N Z J Public Health 2001 25 6 494 497 10.1111/j.1467-842X.2001.tb00310.x 11824981

[B29] Oakley Browne MA Wells JE Scott KM McGee MA The Kessler psychological distress scale in Te Rau Hinengaro: the New Zealand mental health survey Aust N Z J Psychiatry 2010 44 4 314 322 10.3109/00048670903279820 20307164

[B30] Ministry of Health 2006/07 New Zealand Health Survey: content guide 2006 Wellington: Ministry of Health

[B31] Jensen J Spittal M Krishnan V ELSI Short Form: User Manual for a Direct Measure of Living Standards 2005 Wellington: Ministry of Social Development

[B32] Salmond C King P Crampton P Waldegrave C NZiDep: A New Zealand Index of Socioeconomic Deprivation for Individuals 2005 Wellington: University of Otago Wellington and The Family Centre Social Policy Research Unit 10.1016/j.socscimed.2005.08.008 16154674

[B33] Salmond C Crampton P Atkinson J NZDep2006 Index of Deprivation User’s Manual 2007 Wellington: Department of Public Health, University of Otago

[B34] Lumley T Diehr P Emerson S Chen L The importance of the normality assumption in large public health data sets Annu Rev Public Health 2002 23 151 169 10.1146/annurev.publhealth.23.100901.140546 11910059

[B35] Kukutai T The problem of defining an ethnic group for public policy: who is Māori and why does it matter? Soc Pol J New Zeal 2004 23 86 108

[B36] Ajwani S Blakely T Robson B Tobias M Bonne M Decades of Disparity: ethnic mortality trends in New Zealand 1980–1999 2003 Wellington: Ministry of Health and University of Otago

[B37] Curtis E Wright C Wall M The epidemiology of breast cancer in Māori women in Aotearoa New Zealand: implications for screening and treatment N Z Med J 2005 118 1209 U1297 15711630

[B38] Te Rōpū Rangahau Hauora a Eru Pōmare Counting for nothing: understanding the issues in monitoring disparities in health Soc Pol J New Zeal 2000 14 1 16

[B39] Callister P Skin colour: Does it matter? 2008 Wellington: Victoria University

[B40] Ridolfo H Schoua-Glusberg A Analyzing cognitive interview data using the constant comparative method of analysis to understand cross-cultural patterns in survey data Field Meth 2011 23 4 420 438 10.1177/1525822X11414835

[B41] MacLin OH MacLin MK Adams RB, Ambady N, Nakayama K, Shimojo S The role of racial markers in race perception and racial categorization The Science of Social Vision 2010 New York: Oxford University Press 321 346

[B42] Freeman JB Penner AM Saperstein A Scheutz M Ambady N Looking the part: social status cues shape race perception PLoS ONE 2011 6 9 e25107 10.1371/journal.pone.0025107 21977227 PMC3180382

[B43] Paradies YC Cunningham J Development and validation of the measure of indigenous racism experiences (MIRE) Int J Equity Health 2008 7 9 10.1186/1475-9276-7-9 18426602 PMC2359753

[B44] Veenstra G Mismatched racial identities, colourism, and health in Toronto and Vancouver Soc Sci Med 2011 73 1152 1162 10.1016/j.socscimed.2011.07.030 21908088

